# Identification of SMYD2 as a candidate diagnostic and prognostic biomarker for gastric cancer

**DOI:** 10.3389/fonc.2025.1617971

**Published:** 2025-07-24

**Authors:** Sichao Wang, Chuanxi Zhao, Dongmei Li, Qingzhi Liu, Cuiping Mao, Shanshan Ding, Shujun Zhang, Wenjing Shang

**Affiliations:** ^1^ Department of Infectious Disease and Hepatology, The Second Hospital of Shandong University, Jinan, Shandong, China; ^2^ Department of Clinical Laboratory, Shandong Cancer Hospital and Institute, Shandong First Medical University and Shandong Academy of Medical Sciences, Jinan, Shandong, China; ^3^ Department of Radiology, Shandong Cancer Hospital and Institute, Shandong First Medical University and Shandong Academy of Medical Sciences, Jinan, Shandong, China; ^4^ Department of Clinical Laboratory, Qilu Hospital of Shandong University, Jinan, Shandong, China

**Keywords:** gastric cancer, histone modification enzymes, diagnosis, prognosis, SMYD2

## Abstract

**Background:**

Histone modification enzymes (HMEs) are associated with cancer development, treatment response, and prognosis. However, the potential roles of HMEs in gastric cancer (GC) remain unclear. This study aimed to investigate their biological functions and mechanisms in GC, with additional focus on exploring the clinical value of SMYD2.

**Methods:**

We performed integrated analyses of transcriptome profiling and somatic mutation alteration in GC samples from the Cancer Genome Atlas (TCGA) and the Gene Expression Omnibus (GEO) datasets to characterize HMEs alterations in GC. Consensus unsupervised clustering analysis was performed to identify HMEs-associated GC subtypes. Various machine learning methods were employed to construct an HMEs-based diagnostic model for GC. The area under the receiver operating characteristic (ROC) curve (AUC) was used to evaluate model performance. SMYD2 expression in GC tissues was analyzed using TCGA and GEO data and validated by immunohistochemistry (IHC). The association between SMYD2 and the tumor immune microenvironment in GC was evaluated using CIBERSORT, ESTIMATE, and TIDE algorithms. Functional characterization of SMYD2 was performed via SMYD2 knockdown in GC cells.

**Results:**

Most HMEs were up-regulated in GC tissues and exhibited relatively high mutation frequencies. GC patients were stratified into three HMEs-associated subtypes, with cluster 2 (C2) demonstrating significantly better prognosis than C1 and C3. The diagnostic model based on HMEs expression profiles showed robust performance for GC diagnosis. Notably, SMYD2 expression showed positive associations with CD8^+^ T cells, activated CD4^+^ T cells, and M0/M1 macrophages, but negative associations with M2 macrophages, regulatory T cells, stromal score, and TIDE score. Functional assays demonstrated that SMYD2 promoted GC cell proliferation, invasion, and migration *in vitro*.

**Conclusions:**

These findings established SMYD2 is a major oncogene that can serve as a candidate diagnostic and prognostic biomarker for GC.

## 1 Introduction

Globally, an estimated 20 million cancer cases and 9.7 million cancer-related deaths were reported in 2022, with gastric cancer (GC) accounting for 4.9% of new diagnoses and 6.8% of cancer mortality. Ranked as the fifth most prevalent malignancy and the fifth leading cause of cancer death, GC demonstrates multifactorial etiology (1). Established risk factors include pathogenic infections [particularly *Helicobacter pylori* (*H. pylori*)], aging, genetic susceptibility, epigenetic dysregulation, and lifestyle/dietary patterns (2–4). As a Group I carcinogen, *H. pylori* induces chronic gastritis, progressing through gastric intestinal metaplasia and dysplasia to adenocarcinoma (5, 6). Prophylactic *H. pylori* eradication significantly reduces GC incidence in asymptomatic populations (7, 8). Current therapeutic approaches encompass surgical resection, chemotherapy, radiotherapy, and immunotherapy (9). Despite substantial improvements in treatment strategies, the 5-year survival rate remains suboptimal, primarily due to late-stage diagnosis in most patients (10). These clinical challenges underscore the critical need for identifying pivotal molecular markers to advance GC diagnosis and therapeutic development.

Emerging evidence demonstrates that dysregulation of histone methyltransferases (HMTs) plays pivotal roles in tumorigenesis and cancer progression (11–13). HMTs are divided into protein lysine methyltransferases (PKMTs) and protein arginine methyltransferases (PRMTs) (14, 15). As a PKMT family member, SMYD2 (SET and MYND domain-containing protein 2) catalyzes lysine methylation in both histone and non-histone targets (16, 17). SMYD2 primarily functions as an H3K36 methyltransferase that represses histone transcriptional activity, with additional capacity to mediate H3K4 methylation for gene expression regulation (18, 19). Meanwhile, SMYD2 could methylate non-histone proteins, including P53, RB, PTEN, STAT3, p65, HSP90, MAPKAPK3 (20). Epigenetic silencing of tumor suppressors p53 and RB via methylation establishes SMYD2 as a therapeutic target (21). SMYD2 could methylate PTEN at lysine 313, thereby activating PI3K-AKT pathway and driving the proliferation of breast cancer cells (22). SMYD2 could be recruited to substrates of STAT3 and p65 and methylate STAT3 and p65, leading to the proliferation of TNBC cells (23). HSP90 was methylated by SMYD2 at lysine 531 and 574, thereby accelerating the proliferation of cancer cells (24). SMYD2 could also methylate stress response kinase MAPKAPK3 and increased cell proliferation in pancreatic ductal adenocarcinoma (25).

Aberrant expression of SMYD2 is closely associated with several diseases, including cardiovascular diseases and cancer. The SMYD2-HDAC-SRF axis was a critical epigenetic mechanism and regulated vascular smooth muscle cells' phenotypic switching and neointimal hyperplasia (26). SMYD2 overexpression inhibited cell proliferation and migration of vascular smooth muscle and attenuated arterial narrowing in injured vessels via a myocardin-dependent epigenetic regulatory mechanism in mice (27). SMYD2 physically interacted with HNRNPK and mediated lysine monomethylation at K422 of HNRNPK, promoting colorectal cancer angiogenesis by stabilizing EGFL7 mRNA (28). SMYD2 could methylate c-Myc and promote hepatocellular carcinoma progression by reprogramming glutamine metabolism through c-Myc/GLS1 signaling (29). SMYD2 could activate the transcription of PRS7 by binding to its promoter and promote tumorigenesis and metastasis in lung adenocarcinoma (30). In addition, SMYD2 monomethylated non-histone PPARγ and inhibited its nuclear translocation, which facilitated hypoxia-induced pulmonary hypertension (17).

In this study, we used The Cancer Genome Atlas (TCGA) and Gene Expression Omnibus (GEO) datasets to investigate the molecular alterations of HMEs in GC. Moreover, we explored biological functions, diagnostic and prognostic values of HMEs in GC. Furthermore, we focused on SMYD2, which was up-regulated in GC. SMYD2 could regulate multiple tumor-associated signaling pathways. In addition, *in vitro* experiments showed that knockdown of SMYD2 inhibited the proliferation, invasion and migration of GC cells. In conclusion, we proved that SMYD2 was an oncogene in GC and could serve as a potential diagnostic target.

## 2 Materials and methods

### 2.1 Data acquisition

Gene expression data, associated mutations, and clinical data of GC patients were downloaded from TCGA (https://portal.gdc.cancer.gov/). Series matrix file of GSE13195, GSE66229, GSE15459, GSE26253 and GSE84433 were downloaded from the GEO dataset (https://www.ncbi.nlm.nih.gov/geo/). The differential HMEs were determined using limma R package with adjusted *P*-value less than 0.05. A tissue microarray comprising 86 paired GC tumor tissues and adjacent normal specimens, supplemented with 8 additional GC cases, was purchased from Shanghai Qutdo Biotech Company (Shanghai, China). The research related to human use has been complied with all the relevant national regulations, institutional policies and in accordance with the tenets of the Helsinki Declaration, and has been approved by the Ethics Committee of the Shandong Cancer Hospital and Institute (Jinan, China).

### 2.2 Cell culture

AGS cells were obtained from the Cell Bank of the Chinese Academy of Science (Shanghai, P.R. China). MKN-45 cells were obtained from the National Infrastructure of Cell Line Resource (Beijing, P.R. China). AGS cells were cultured in Ham’s F12-medium (HyClone, Logan, UT, USA) supplemented with 12% fetal bovine serum (Gibco, Carlsbad, CA, USA). MKN-45 cells were cultured in RPMI-1640 medium (Gibco) containing 10% fetal bovine serum. All cell lines were incubated under standardized conditions in a humidified atmosphere (37°C, 5% CO_2_) using a Thermo Fisher Scientific HERAcell 150i incubator (Waltham, MA, USA). All cells were authenticated by short tandem repeat profiling, and tested free from *Mycoplasma*.

### 2.3 Consensus clustering analysis of HMEs

A total of 74 HMEs, listed in **Supplementary Table 1**, were obtained from previous publications (10). 34 shared HMEs between TCGA and GEO GC expression datasets were selected for clustering analysis using the “ConsensusClusterPlus” R package. Three novel GC subtypes were identified based on the cumulative distribution function curve parameters, which exhibited a gradual and smooth increase.

### 2.4 KEGG and GO

Functional enrichment analysis for KEGG and GO terms was performed using the clusterProfiler R package.

### 2.5 Immune infiltration analysis

Stromal score, Immune score, and ESTIMATE score for each GC patient were calculated from gene expression profiles using the ESTIMATE R package (31). Immune cell composition in GC tissues was quantified using the CIBERSORT algorithm (32).

### 2.6 Tumor immune dysfunction and exclusion score and microsatellite instability status analysis

The TIDE scores and the Microsatellite Instability status of GC samples were assessed utilizing the TIDE online platform (http://tide.dfci.harvard.edu/login/) (33, 34).

### 2.7 Transfection assays

Lipofectamine 2000 (Invitrogen, Carlsbad, CA, USA) was used to transfect siRNAs into GC cells. Cells were collected 72 hours post-transfection. RT-PCR was used to verify the transfection efficiency. The siRNA sequences were listed as follows: si-SMYD2-1 5’-CUCGGAGACUGUAAGACUATT-3’ and si-SMYD2-2 CCCAACGGAAGAUAGAAAUTT-3’.

### 2.8 *H. pylori* culture

*H. pylori* strains 26695 and 11637 were cultured in Brucella broth supplemented with 5% fetal bovine serum at 37°C under microaerophilic conditions. AGS and MKN-45 cells were infected with *H. pylori* at varying concentrations and collected at specified time points.

### 2.9 RNA extraction and RT-PCR

Trizol reagent (Invitrogen, Carlsbad, CA, USA) was used to extract RNAs following the manufacturer’s instructions. RT reagent Kit gDNA Eraser (Takara, Shiga, Japan) was employed to reverse mRNAs to cDNA. Finally, SYBR Green (Takara) was used for qRT-PCR of cDNAs. Primer sequences were listed as follows: SMYD2 forward 5’-TGTGTTTGAGGACAGTAACGTG-3’ and reverse 5’-GAGGGAGTACAAAGGATAGTGC-3’; β-actin forward 5’ - AGTTGCGTTACACCCTTTCTTG - 3’ and reverse 5’ - CACCTTCACCGTTCCAGTTTT - 3’.

### 2.10 Colony formation and CCK8 assay

500 cells per well with different treatments were plated in 6-well plates and cultured for 1–2 weeks. Colonies were fixed with methanol and stained with Giemsa solution. Colonies containing > 50 cells were counted for analysis.

1200 cells per well with different treatments were plated in 96-well plates and cultured for 24, 48, or 72 hours for CCK8 assay. CCK8 reagent (Med Chem Express, Monmouth Junction, NJ, USA) was added to the conditioned medium and incubated for 3 hours. Absorbance was measured at 450 nm using a spectrophotometer. Both assays were performed in triplicate.

### 2.11 Wound healing and transwell migration assays

For wound healing assays, cells (5 × 10^5^) with different treatments were seeded into 12-well plates and grown to confluence. A sterile pipette tip was used to create a linear scratch. Wound width was measured at 0 and 48 hours. Wound closure rate was calculated as: (wound width at 0 hours – wound width at 48 hours)/wound width at 0 hours × 100%. An inverted phase-contrast microscope was used to view the cells and photograph.

For migration assays, cells (5 × 10^4^) were seeded into the upper chamber of a Matrigel-coated transwell insert. The medium containing 20% FBS was added to the lower chamber. After 48 hours, methanol and crystal violet staining solution were used to fix and stain the cells respectively.

### 2.12 IHC for tissue microarray

Tissue microarray on glass slides was subjected to deparaffination and dehydration. Then, the array was subjected to epitope retrieval. Next, the array was treated with H_2_O_2_ and blocked in goat serum for 30 minutes. Primary antibody of SMYD2 was used to incubate the array at 4°C overnight. The array was incubated with the corresponding secondary antibody on the second day. Finally, a DAB staining kit (Vector Laboratories, Burlingame, CA, USA) was used to stain. Images were used to assess the IHC score. The intensity of positive staining was scored as follows: 0 (negative), 1 (weak), 2 (moderate) and 3 (strong). A scale from 0 to 3 was used to score the proportion of positively stained cells: 0 (0%), 1 (<25%), 2 (25-75%) and 3 (>75%). Final IHC score = Intensity score × Proportion score.

### 2.13 Western blotting

Lysis buffer supplemented with protease inhibitors was used to extract proteins from cells. The lysates were resolved by SDS-PAGE. Protein was then transferred to PVDF membranes, which were blocked with 5% non-fat milk powder and subsequently incubated with primary antibodies overnight at 4°C. Following this, PVDF membranes were incubated with appropriate secondary antibodies. Signals were detected using an ECL detection reagent (Millipore, St. Louis, MO, USA). The primary antibody used was SMYD2 (D14H7; Cell Signaling Technology, Danvers, MA, USA).

### 2.14 Statistical analysis

Data were presented as mean ± SEM from three independent experiments. Mann-Whitney *U*-test or Student’s *t*-test was used to compare variables between experimental group and control group by GraphPad PRISM version 9 (San Diego, CA, USA). SPSS version 23.0 (IBM, Armonk, NY, USA) was used to analyze clinical data. *P* < 0.05 was considered statistically significant.

## 3 Results

### 3.1 Genetic and transcription alterations of HMEs in GC

**Figure 1:**
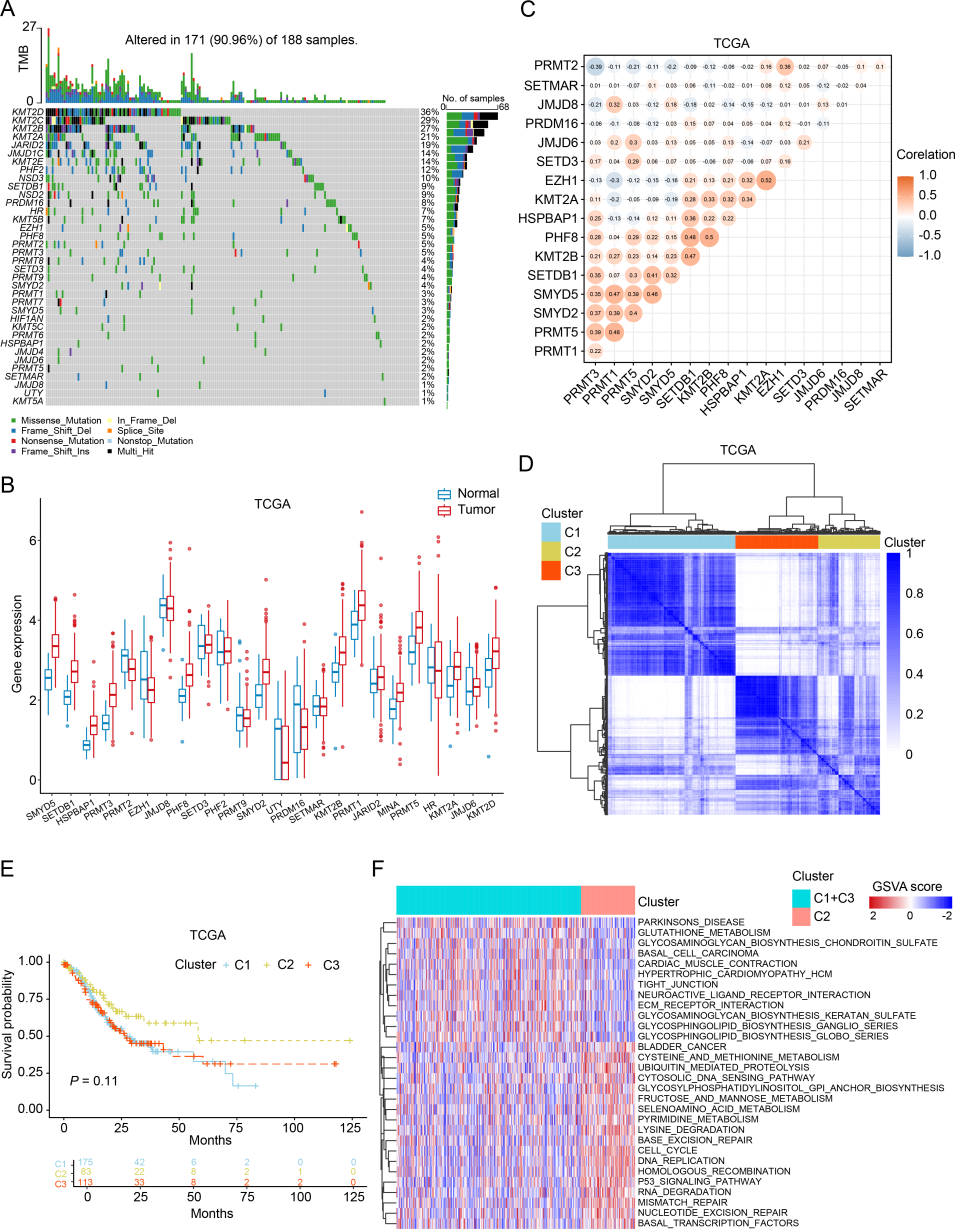
Genetic, transcriptional alterations, and molecular subtypes of HMEs in GC. **(A)** MAFtool exhibited the incidence of somatic mutations of HMEs in 188 GC patients from the TCGA dataset. **(B)** Differential expression of HMEs between GC tissues and paired para-carcinoma tissues. The data were obtained from the TCGA dataset. **(C)** Correlation analysis of HMEs. **(D)** Identification of three molecular subtypes in TCGA GC samples via consensus clustering analysis. **(E)** Survival analysis among C1, C2, and C3. Kaplan-Meier plot and log-rank tests were conducted. **(F)** GSVA enrichment analysis comparing subtypes C2 versus C1+C3. Red indicates pathway enrichment; blue indicates depletion.

In order to investigate the association between HMEs and GC, we performed a comprehensive analysis of somatic mutation frequencies for 74 HMEs using TCGA-STAD data. The results indicated a relatively high mutation frequency in GC samples. In detail, 171 GC samples (90.96%) had HME mutations. Among these, *KMT2D* exhibited the highest mutation frequency (36%), followed by *KMT2C*, *KMT2B*, *KMT2A*, *JARID2*, *JMJD1C*, *KMT2E*, *PHF2*, *NSD3*, *SETDB1*, *NSD2*, *PRDM16* and *HR* (**Figure 1A**). Next, we compared expression of HMEs between GC tissues and adjacent normal samples from the TCGA cohort. 24 HMEs were differentially expressed, with 15 up-regulated and 9 down-regulated genes in GC (**Figure 1B**). Similar trends were observed in the GSE13195 and GSE66229 datasets (**Supplementary Figure 1A**). Correlation analysis revealed moderately correlated HMEs expression patterns between TCGA and GEO datasets (**Figure 1C**; **Supplementary Figure 1B**). We categorized GC patients into three subtypes based on the expression profiles of HMEs using a consensus clustering algorithm (**Figure 1D**; **Supplementary Figures 2A, B**). Survival analysis indicated that patients with GC in cluster 2 (C2) had a higher survival probability than those in cluster 1 (C1) and cluster 3 (C3) (**Figure 1E**; **Supplementary Figure 2C**). To identify subtype-associated pathways, gene set variation analysis was performed comparing C1 + C3 with C2. The results showed that C1+C3 was significantly enriched in cell cycle and *p53* pathways (**Figure 1F**). These findings collectively highlighted the critical regulatory role of HMEs in GC tumorigenesis and progression.

### 3.2 The diagnostic performance of HMEs in GC

**Figure 2:**
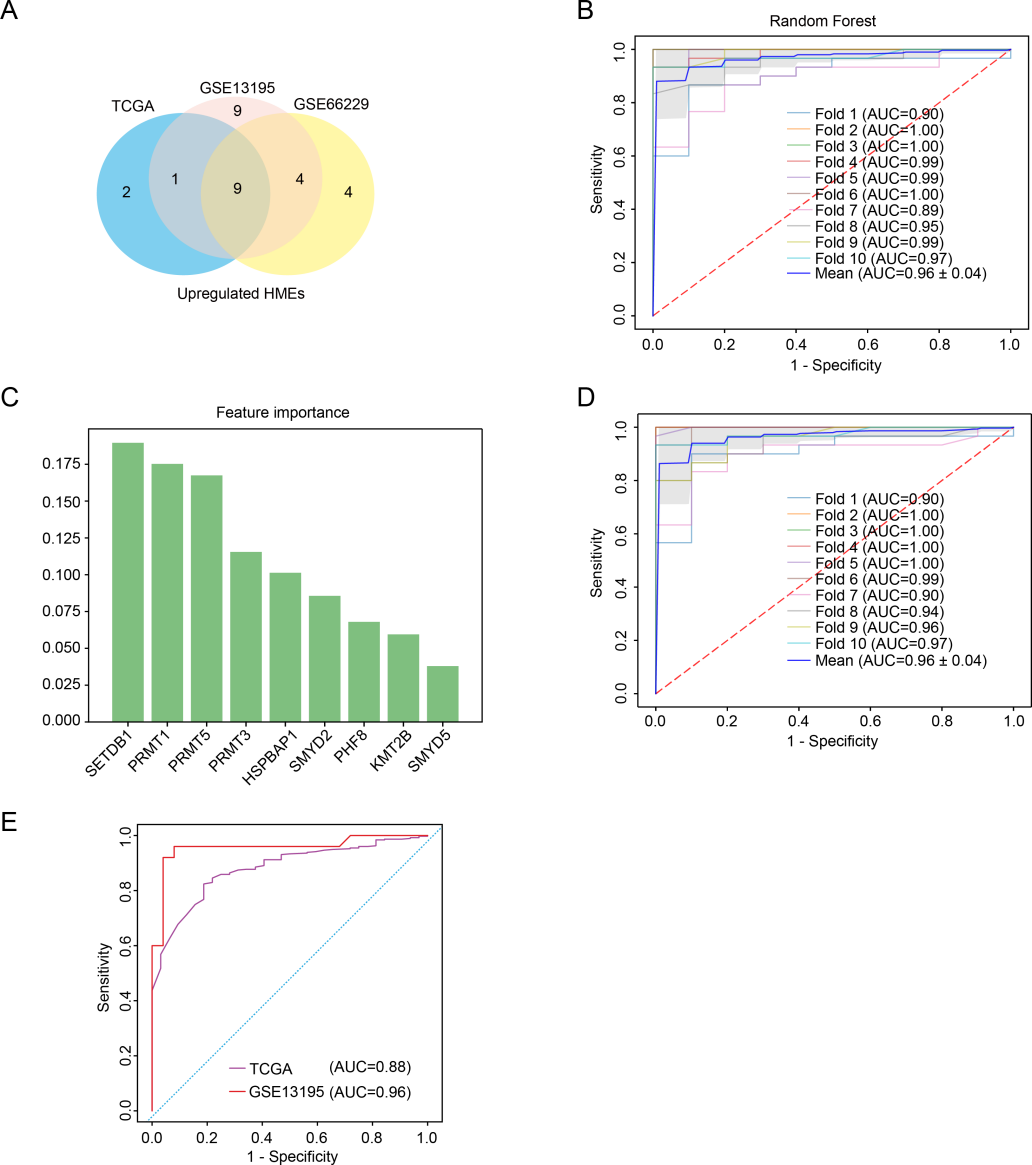
Characterization of key HMEs in GC. **(A)** Venn diagram identifying common upregulated HMEs across GSE13195, GSE66229 and TCGA datasets. **(B)** ROC curve assessing the diagnostic potential of 9 upregulated HMEs using random forest with ten-fold cross-validation. **(C)** Feature importance ranking of the 9 upregulated HMEs derived from the random forest model. **(D)** ROC curve evaluating the diagnostic potential of the 6 upregulated HMEs using ten-fold cross-validation. **(E)** Validation of the 6-HME signature’s diagnostic performance in TCGA GC data and GSE13195.

We have found that the expression of some HMEs was different in GC tissues compared to para-carcinoma tissues. To evaluate their diagnostic potential, we systematically analyzed HMEs expression profiles in TCGA, GSE13195 and GSE66229 datasets, respectively. Consistent intersection analysis revealed 9 consistently up-regulated and 1 down-regulated HME across all datasets (**Figure 2A**; **Supplementary Figure 3A**). We used 9 up-regulated HMEs to train the diagnostic model with ten-fold cross-validation using Random Forest, Logistic Regression, Support Vector Machine, Naive Bayes, Linear Discriminant Analysis, Bagging, and Gradient Boosting Decision Tree (**Supplementary Figure 3B**). Notably, all models demonstrated high diagnostic accuracy with AUC values ranging from 0.95 to 0.96 (**Figure 2B**). Feature selection via Random Forest reduced the biomarker set to six HMEs using an importance threshold of 0.075 (**Figure 2C**). The simplified 6-HME model maintained comparable performance with AUC 0.96 (**Figure 2D**). Validation in TCGA and GSE13195 cohorts confirmed robust diagnostic capability, achieving AUC values of 0.88 and 0.96 respectively (**Figure 2E**). Collectively, these findings demonstrated that HME-based biomarkers exhibit strong potential for gastric cancer diagnosis.

### 3.3 SMYD2 overexpression in GC patients associated with poor prognosis

**Figure 3:**
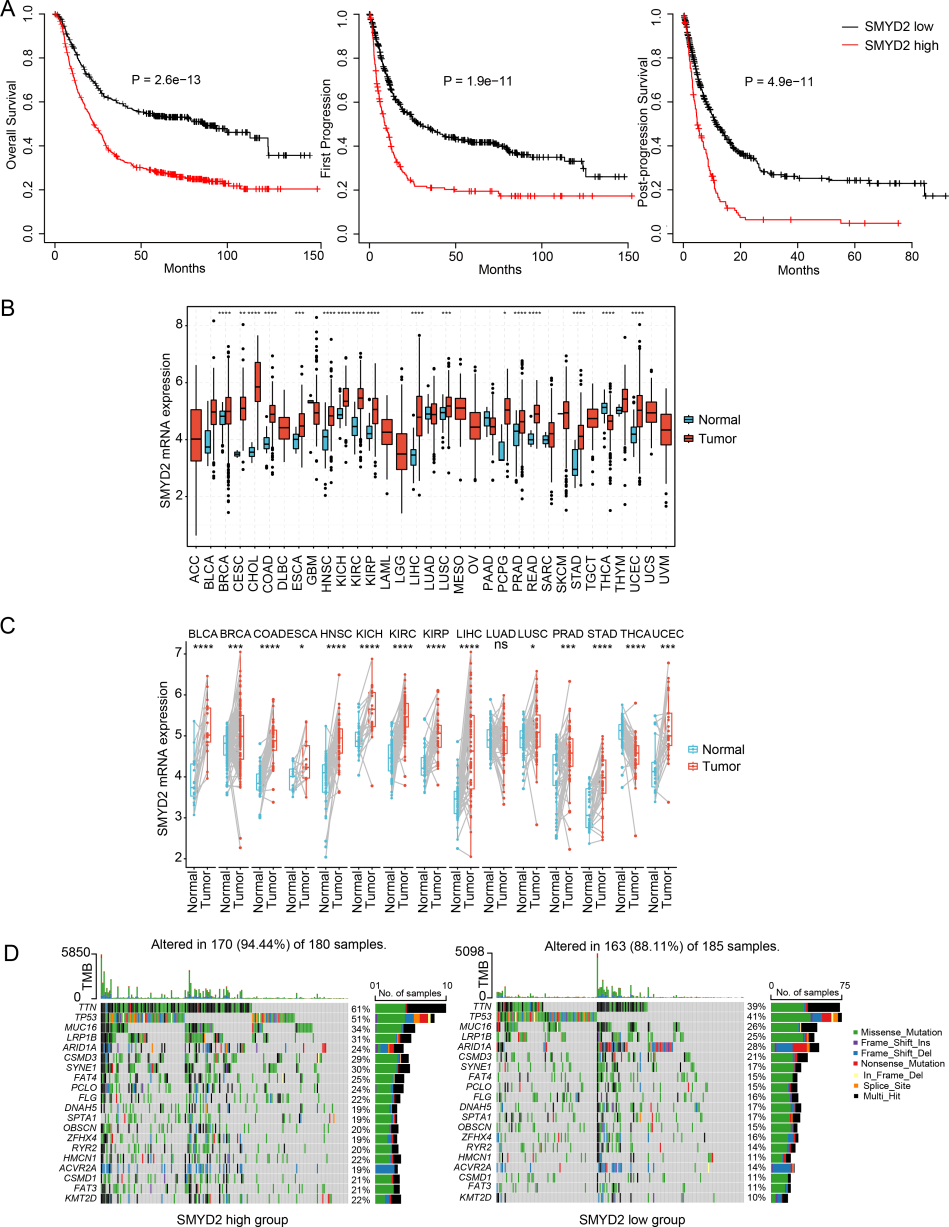
Expression and prognosis significance of SMYD2 in GC. **(A)** Kaplan-Meier survival analysis comparing survival in GC patients stratified by SMYD2 expression. **(B)** SMYD2 expression levels across tumor tissues versus normal tissues based on TCGA dataset. **(C)** SMYD2 expression in paired GC tumor tissues and adjacent normal tissues based on TCGA dataset. **(D)** Waterfall plot depicting somatic mutation profiles in high versus low SMYD2 groups. *P < 0.05, **P < 0.01, ***P < 0.001, ****P < 0.0001.

To identify pivotal regulators of GC development, we focused on the six HMEs including SETDB1, PRMT1, PRMT5, PRMT3, HSPBAP1 and SMYD2. There are fewer studies on SMYD2 than the other genes. Notably, elevated SMYD2 expression correlated significantly with poorer patient prognosis (**Figure 3A**). SMYD2 was also up-regulated across multiple cancer types (**Figure 3B**). Comparative analysis of paired tumor and para-carcinoma tissues showed SMYD2 overexpression across most cancer types, except for lung adenocarcinoma (**Figure 3C**). Furthermore, SMYD2 expression was positively related to tumor mutation burden in GC (**Supplementary Figure 4A**). The high-SMYD2 group exhibited higher mutation counts (**Figure 3D**) and increased mutational co-occurrence compared to the low-SMYD2 group (**Supplementary Figure 4B**).

### 3.4 Function landscape of SMYD2 in GC

**Figure 4:**
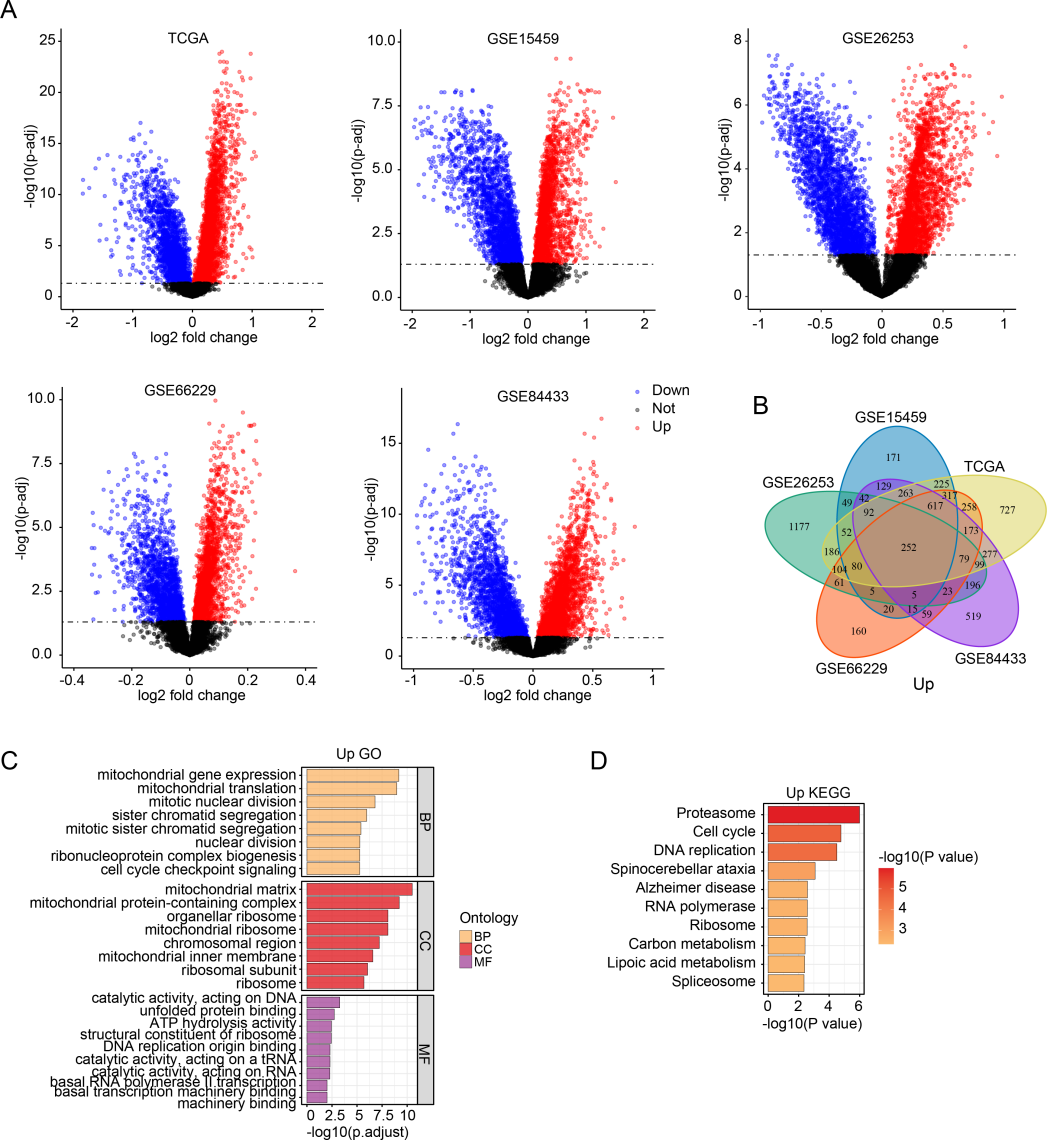
SMYD2-associated genes and pathway enrichment. **(A)** Volcano plots of differentially expressed genes between SMYD2-high and SMYD2-low groups across five datasets (TCGA, GSE15459, GSE26253, GSE66229, GSE84433). **(B)** Venn diagram identifying common upregulated genes in SMYD2-high group across TCGA, GSE15459, GSE26253, GSE66229 and GSE84433 datasets. **(C, D)** GO **(C)** and KEGG **(D)** pathway enrichment analyses of the 252 consistently upregulated genes in SMYD2-high group.

To identify SMYD2-associated genes and pathways, we performed comparative transcriptomic analysis of TCGA and GEO datasets, analyzing differentially expressed genes (adjusted p-value < 0.05) between SMYD2-high and SMYD2-low expression groups (stratified by median expression cutoff) (**Figure 4A**). Venn diagram analysis identified 252 consistently up-regulated genes (**Figure 4B**) and 277 down-regulated genes (**Supplementary Figure 5A**). GO enrichment analysis revealed that up-regulated genes were predominantly associated with mitochondrial function, mitotic processes and cell cycle checkpoint pathways (**Figure 4C**). Concurrently, KEGG pathway analysis demonstrated significant enrichment of up-regulated genes in proteasome activity, cell cycle regulation and DNA replication pathways (**Figure 4D**). For down-regulated genes, both GO and KEGG analyses showed primary enrichment in biological processes related to cell-substrate adhesion, cytoskeletal organization, and focal adhesion pathways (**Supplementary Figures 5B, C**). These collective findings suggest that SMYD2 may modulate signaling networks governing tumor cell proliferation and metastatic processes.

### 3.5 Association between SMYD2 and immune infiltration in GC

**Figure 5:**
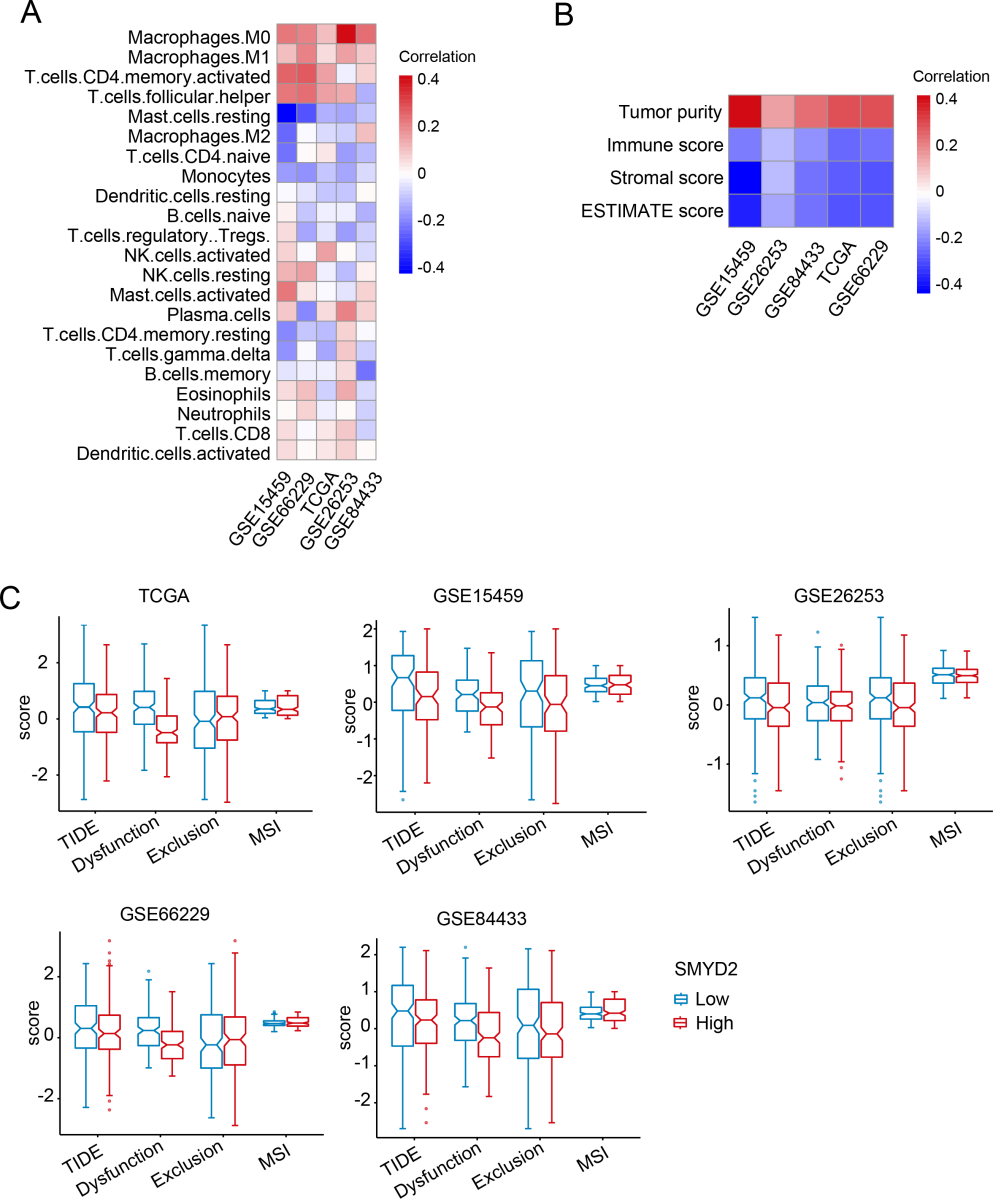
Correlation of SMYD2 with tumor immunity in GC. **(A)** Association between SMYD2 expression levels and immune cell infiltration across multiple GC expression datasets. **(B)** Association between SMYD2 expression and immune/stromal/ESTIMATE scores. **(C)** Correlation of SMYD2 expression with Tumor Immune Dysfunction and Exclusion (TIDE) scores, T cell dysfunction, T cell exclusion, and microsatellite instability (MSI) in TCGA and GEO datasets.

To explore the association between SMYD2 expression and tumor immune infiltration in GC patients, we performed CIBERSORT analysis in TCGA and GEO datasets. SMYD2 expression positively correlated with CD8^+^ T cells, activated CD4^+^ T cells, and M0/M1 macrophage infiltration, but negatively correlated with M2 macrophages and regulatory T cells (Tregs) (**Figure 5A**). Subsequent analysis revealed SMYD2 positively correlated with tumor purity while inversely correlating with immune score, stromal score, and ESTIMATE score (**Figure 5B**). Using the TIDE algorithm (34), we evaluated immunotherapy response potential, where lower TIDE scores indicate greater predicted benefit. High SMYD2 expression associated with reduced TIDE, dysfunction, and exclusion scores (**Figure 5C**), suggesting enhanced immunotherapy sensitivity in SMYD2-high GC patients. These findings implicate SMYD2 as a predictive biomarker for immunotherapy efficacy in GC.

### 3.6 SMYD2 expression was elevated in GC tissues

**Figure 6:**
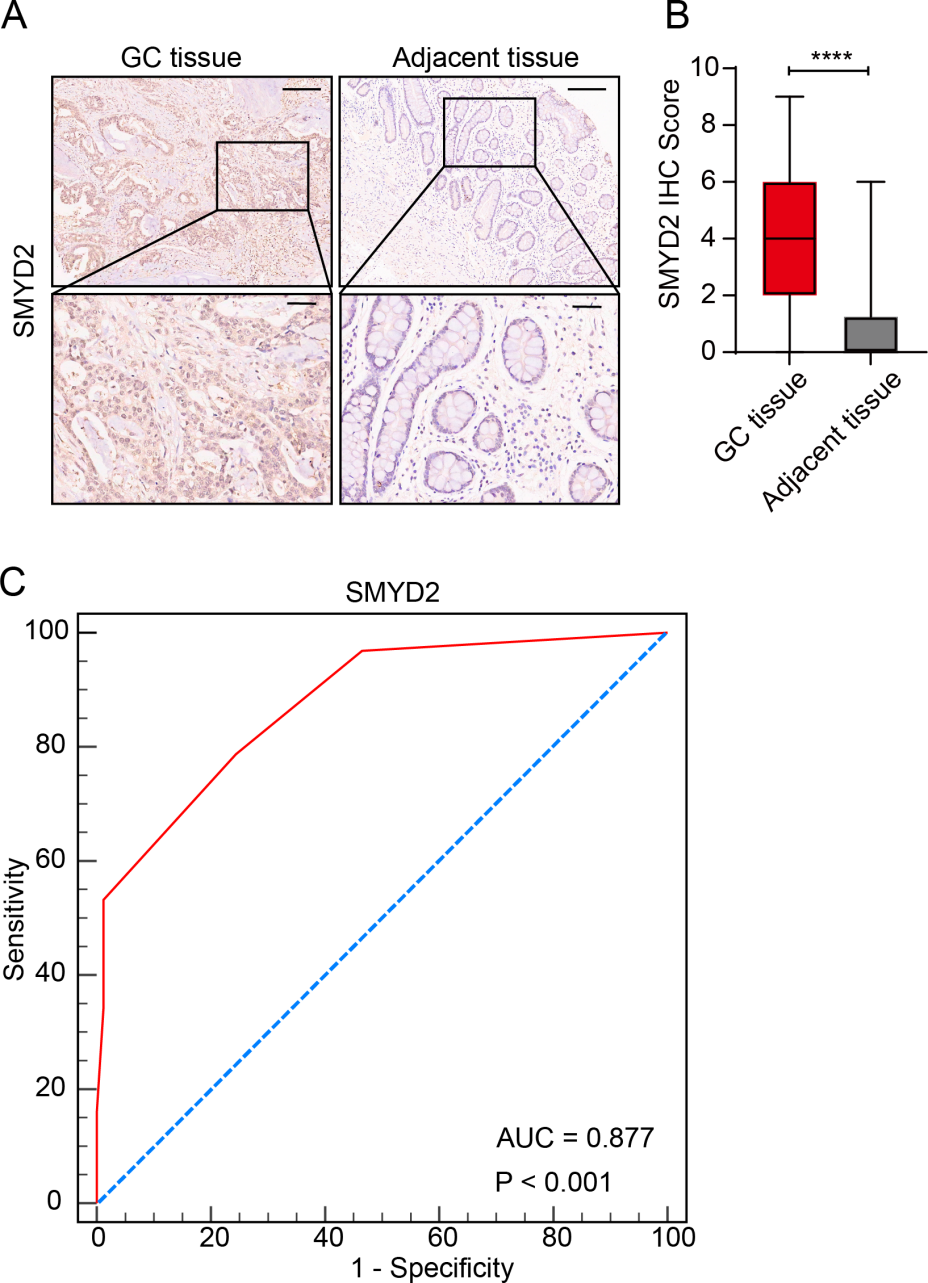
Validation of SMYD2 expression and diagnostic potential in GC tissues. **(A)** Representative IHC staining of SMYD2 in GC tissues and adjacent non-tumor tissues. Scale bars: 200 μm (insets: 50 μm). **(B)** Quantification of SMYD2 IHC staining intensity demonstrating significantly elevated expression in GC tissues compared to adjacent tissues (****P < 0.0001, unpaired Student’s *t*-test). **(C)** ROC curve evaluating the diagnostic performance of SMYD2 IHC score.

We measured SMYD2 expression in human GC samples and adjacent normal samples using a tissue microarray. Results showed that SMYD2 expression was significantly elevated in GC samples (**Figures 6A, B**). ROC curve analysis revealed SMYD2’s discriminative capacity for GC diagnosis, yielding an AUC of 0.877 (**Figure 6C**). Association analysis between SMYD2 expression and clinicopathological characteristics revealed significant correlation with Borrmann subtype (*P* = 0.005), while showing non-significant associations with other parameters (**Supplementary Table 2**).

### 3.7 SMYD2 promoted GC cell proliferation, invasion and metastasis

**Figure 7:**
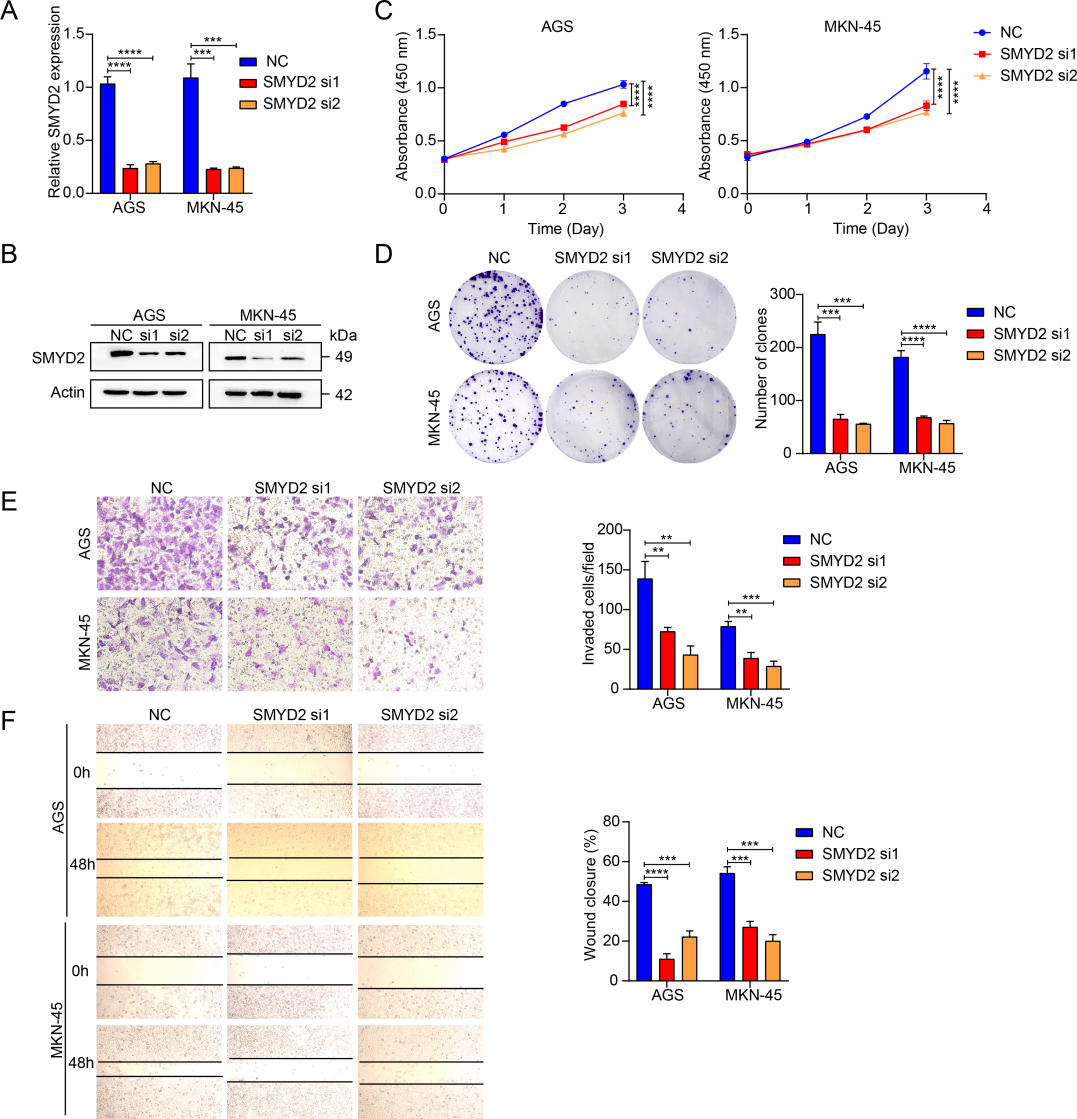
SMYD2 facilitates GC cell proliferation and metastasis. **(A)** SMYD2 mRNA expression following transfection with SMYD2-targeting siRNAs. **(B)** SMYD2 protein expression following transfection with SMYD2-targeting siRNAs. **(C)** CCK8 assay after cells transfected with SMYD2 siRNAs. **(D)** Colony formation assay after cells transfected with SMYD2 siRNAs. **(E)** Transwell penetration assays in SMYD2-knockdown AGS and MKN-45 cells. **(F)** Cell migration assessed by wound healing assay in SMYD2-knockdown AGS and MKN-45 cells. **P <0.01, ***P < 0.001, P < 0.0001, by Student’s *t*-test.

We next explored the functions of SMYD2, and we performed siRNA-mediated knockdown in GC cells (**Figures 7A, B**). SMYD2 suppression significantly attenuated cellular proliferation, as evidenced by colony formation and CCK-8 assays (**Figures 7C, D**). Furthermore, SMYD2 silencing markedly impaired invasion and migration capacities in Transwell and wound healing assays, respectively (**Figures 7E, F**). These results reiterated the positive role of SMYD2 in tumorigenesis and progression of GC *in vitro*.

### 3.8 *H. pylori* promoted SMYD2 expression

**Figure 8:**
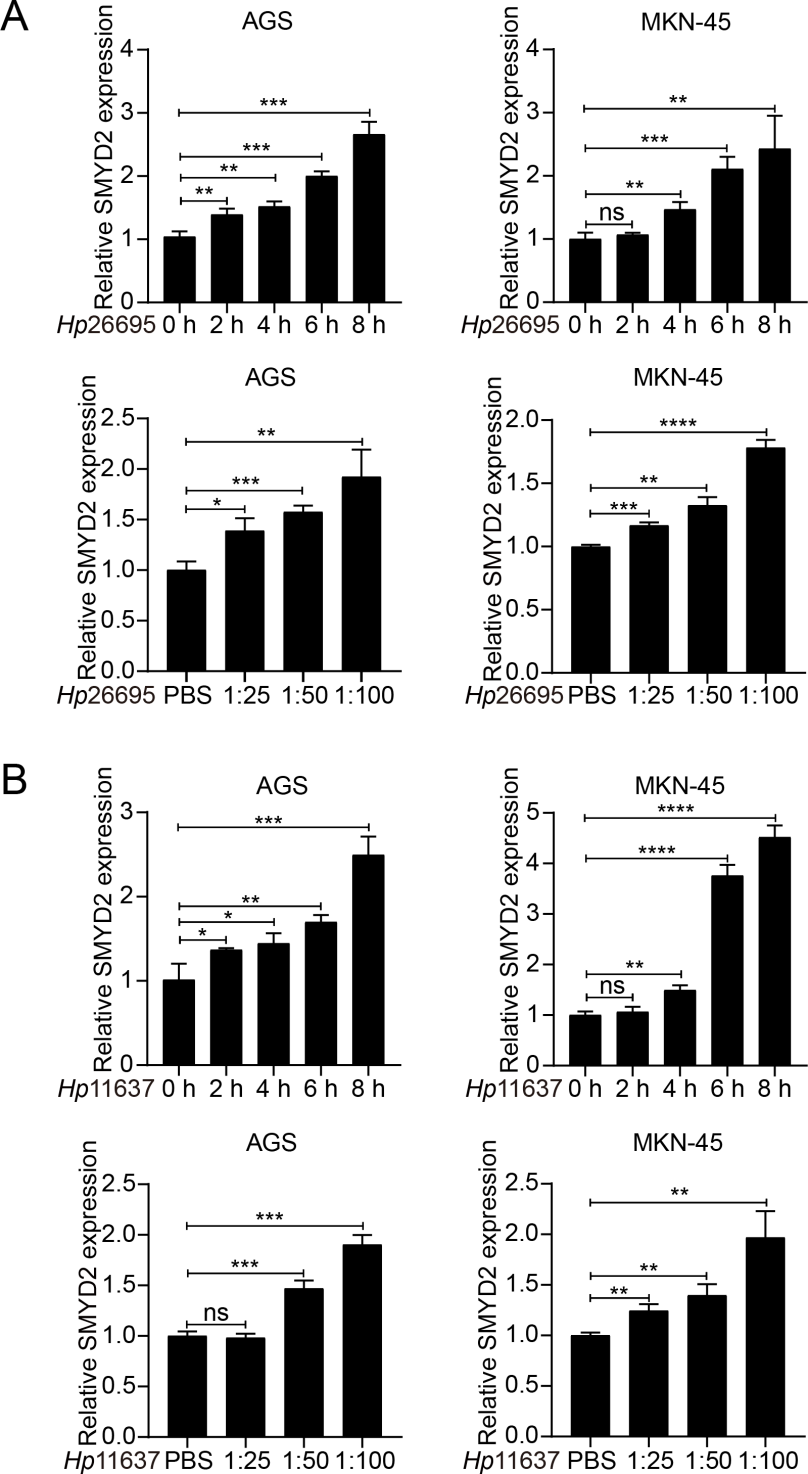
*H. pylori* infection induces SMYD2 expression. **(A)** RT-qPCR analysis of SMYD2 mRNA levels in AGS and MKN-45 cells infected with *H. pylori* strain 26695 at different MOIs and different time points. **(B)** RT-qPCR analysis of SMYD2 mRNA expression in AGS and MKN-45 cells infected with *H. pylori* strain 11637 at different MOIs and different time points. *P < 0.05, **P < 0.01, ***P < 0.001, ****P < 0.0001, by Student’s *t*-test.

Infection of *H. pylori* is a well-known risk factor for GC (35, 36). Therefore, we explored whether SMYD2 expression was regulated by *H. pylori*. We detected SMYD2 expression after *H. pylori* strains (26695 and 11637) infection in AGS and MKN-45 cells. The results showed that SMYD2 expression was significantly up-regulated after infection with *H. pylori* at different MOIs. Meanwhile, SMYD2 expression was also increased at different time points after infection with *H. pylori* (**Figures 8A, B**). This finding was corroborated by bioinformatics analysis of GEO datasets, demonstrating significantly elevated SMYD2 levels in *H. pylori*-positive clinical specimens compared to uninfected controls (**Supplementary Figure 6A**).

## 4 Discussion

HMTs are post-translational modification-related enzymes, whose abnormal expression could lead to several diseases, such as cancer (37–39). HMTs are divided into PKMTs and PRMTs based on their methyltransferase activity on lysine or arginine residues (40). SMYD2 is a PKMT and possessed five structurally distinct domains, including S-sequence, MYND domain, insertion SET domain, cysteine-rich post-SET domain and tetratricopeptide repeat domain (21). The enzymatic activity of SMYD2 mainly relies on the post-SET domain with complete functional ablation observed upon domain deletion (41). SMYD2 plays an important role in various diseases, including cardiac disease and cancers (42). High expression of SMYD2 is closely associated with the poor prognosis of patients.

Our pan-cancer analysis of TCGA data revealed consistent and significant SMYD2 upregulation across multiple malignancies including GC, breast cancer, colorectal adenocarcinoma, renal cell carcinoma, and prostate cancer (**Figures 3B, C**), validating and extending prior reports (16, 43–45). Tissue microarray analysis identified a significant association between SMYD2 expression and Borrmann subtype (*P* = 0.005) but not TNM staging (**Supplementary Table 2**), a discrepancy potentially attributable to current sample size limitations that warrants investigation in future stage-stratified cohorts. Furthermore, we also found SMYD2 as a highly promising diagnostic biomarker for GC (AUC = 0.877), though its successful clinical implementation will require: large-scale multicenter validation of key diagnostic parameters including accuracy, specificity, inter-laboratory reproducibility, and systematic cost-benefit analyses comparing SMYD2 testing against existing diagnostic paradigms.

Immunotherapeutic implications of SMYD2 were evaluated using TIDE scoring in TCGA and GEO datasets, revealing significant clinical relevance (**Figure 4C**). SMYD2 expression showed a positive correlation with tumor purity but inverse relationships with immune, stromal, and ESTIMATE scores (**Figure 5B**). Notably, elevated SMYD2 expression was associated with reduced TIDE scores (**Figure 5C**), collectively suggesting that GC patients with high SMYD2 expression might demonstrate enhanced responsiveness to immunotherapy. This observation aligns with previous reports that SMYD2 modulates the cGAS-STING pathway to regulate CD8^+^ T cell activation and antitumor immunity (46). However, the precise mechanisms by which SMYD2 influences immune infiltration in gastric cancer remain to be fully elucidated.

Functional analysis confirmed that SMYD2 knockdown suppressed proliferation, invasion and migration of GC cell *in vitro* (**Figures 7C–F**). These results were consistent with those from previous studies (47, 48). Xu et al. demonstrated that LncRNA DLEU1 recruits SMYD2 to upregulate APOC1 expression, thereby enhancing GC cell proliferation and glycolytic activity (47). Similarly, Liu et al. identified that neutrophil extracellular traps promote GC metastasis through SMYD2 modification (48). While these studies implicate SMYD2 in gastric carcinogenesis, the precise molecular mechanisms underlying its oncogenic functions in GC remain incompletely characterized, warranting further mechanistic investigation. *H. pylori* is the strongest risk factor for gastritis and gastric adenocarcinoma (49–51). We detected SMYD2 expression after infection with *H. pylori* and found that *SMYD2* expression was remarkably increased with *H. pylori* infection in different MOIs and different time points (**Figures 6A, B**). To our knowledge, this represents the first report linking *H. pylori* infection to SMYD2 dysregulation. The molecular pathways mediating this relationship require elucidation in future studies.

In summary, this study establishes SMYD2 as both a pivotal oncogenic driver and a promising diagnostic biomarker in gastric carcinogenesis. The identification of the *H. pylori*-SMYD2 axis provides novel mechanistic insights into infection-associated GC development.

## 5 Conclusions

In this study, we found that SMYD2 was highly expressed in GC, which was associated with poor prognosis. SMYD2 promoted the occurrence and progression of GC. Therefore, SMYD2 had an oncogenic role in GC and can be regarded as a potential therapeutic target.

## Data Availability

The original contributions presented in the study are included in the article/**Supplementary Material**. Further inquiries can be directed to the corresponding author.
