# Supplementary material for: Identification of SMYD2 as a candidate diagnostic and prognostic biomarker for gastric cancer
Source: Front Oncol. 2025 Jul 24;15:1617971. doi: 10.3389/fonc.2025.1617971 (PMC12328180; doi:10.3389/fonc.2025.1617971)
Supplement: Supplementary file 1 [file DataSheet1.docx]

Supplementary Material

**Supplementary Figure 1.** **Expression alteration of HMEs in GC. (A)** The expression profiles of HMEs in gastric cancer versus adjacent normal tissues. The data were obtained from GSE13195 and GSE66229. **(B)** Correlation analysis of HMEs across GSE13195, GSE15459, GSE66229, GSE84433 and GSE26253 datasets.


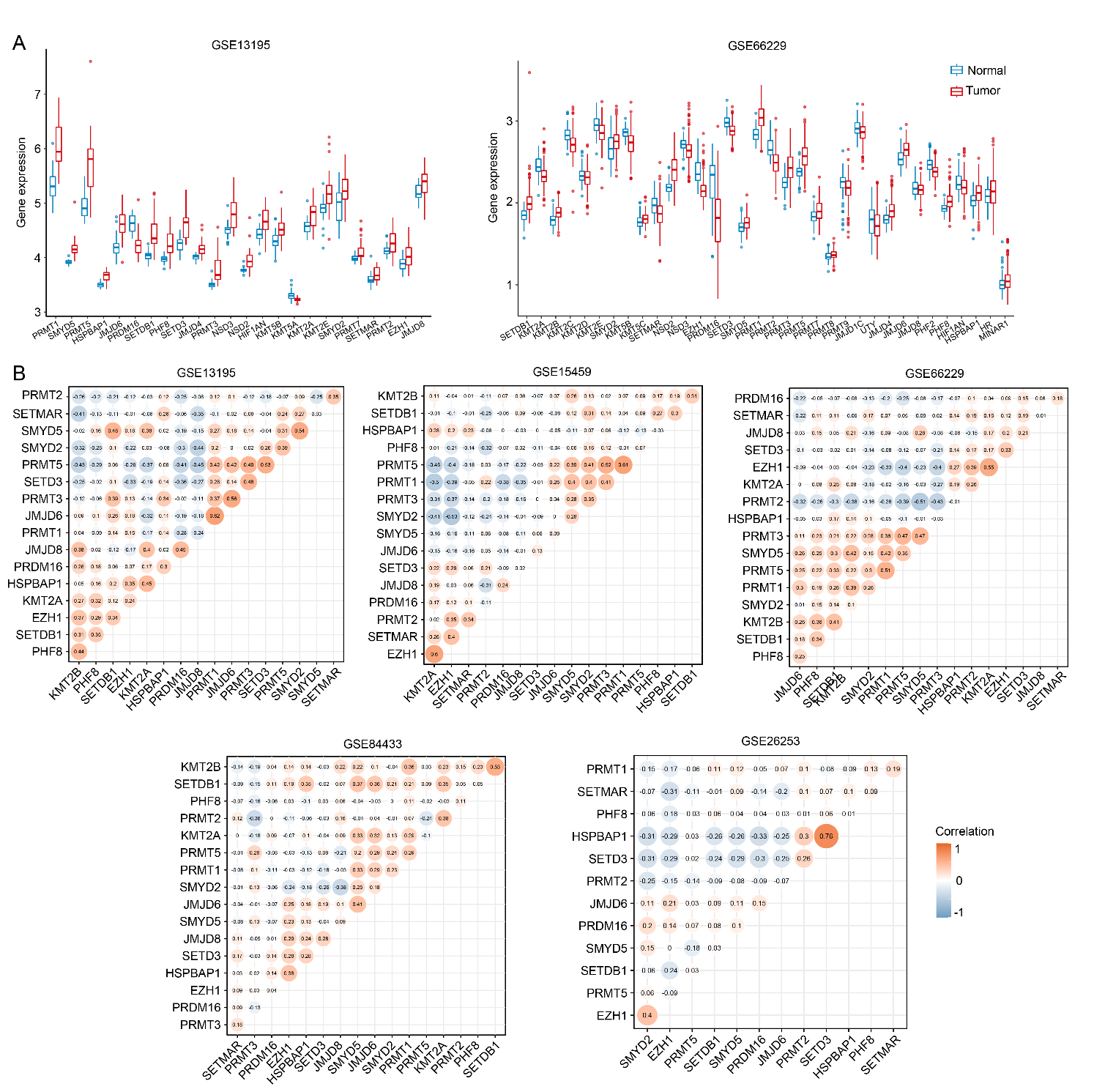


**Supplementary Figure 2.** **Consensus clustering analysis of GC subtypes. (A)** Delta area curves for determining optimal cluster number in TCGA, GSE15459, GSE26253, GSE66229 and GSE84433 cohorts. **(B)** Consensus matrix heatmap identifying three molecular subtypes. The data were obtained from GSE15459, GSE26253, GSE66229 and GSE84433 datasets. **(C)** Significant survival difference between C1+C3 and C2 by Kaplan-Meier analysis.


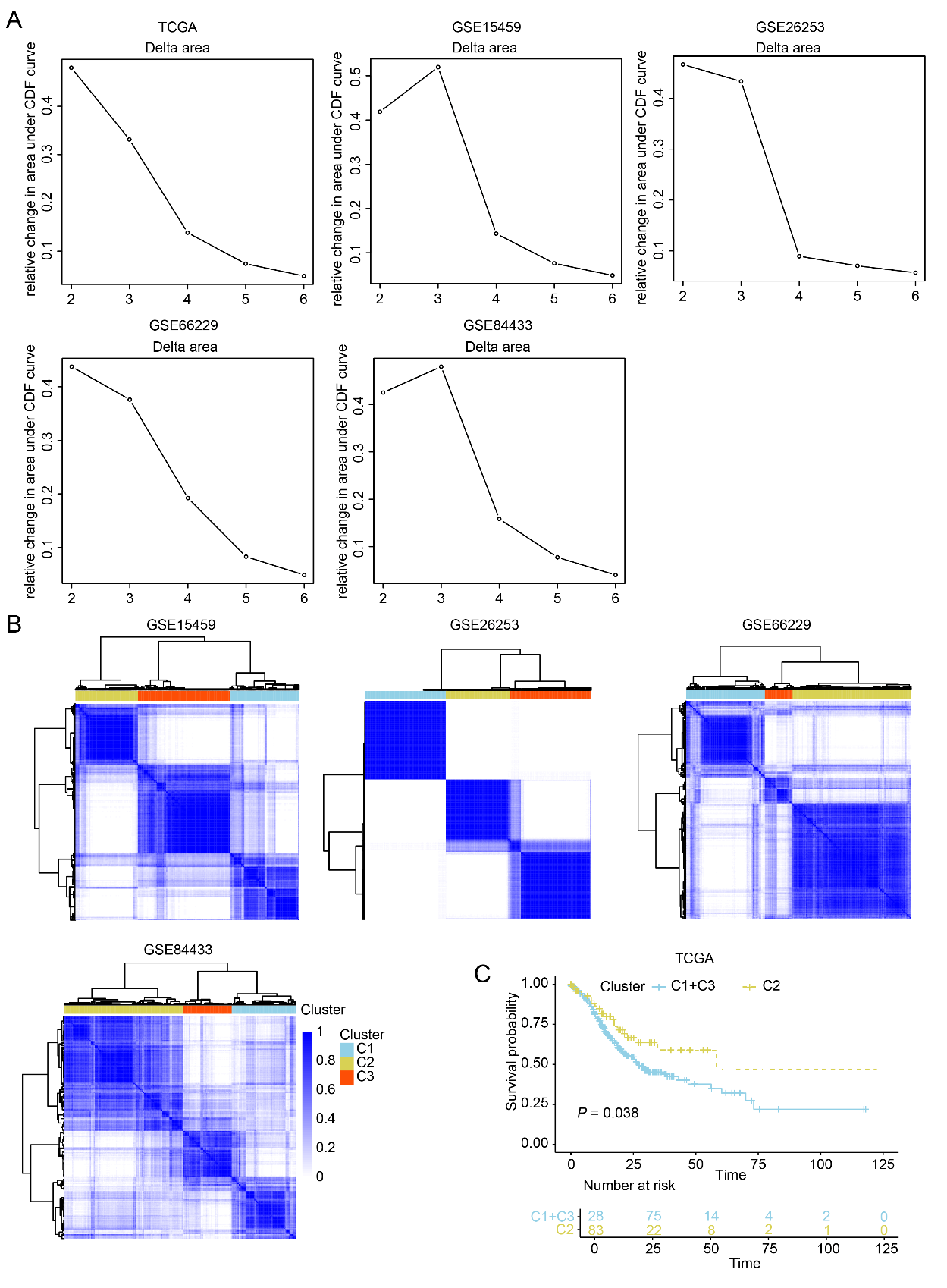


**Supplementary Figure 3.** **Diagnostic performance of HME-based biomarkers in GC. (A)** Venn diagram showing the overlapping downregulated HMEs across GSE13195, GSE66229, and TCGA datasets. **(B)** ROC curves demonstrating diagnostic accuracy of 9 upregulated HMEs using six machine learning algorithms with 10-fold cross-validation: Logistic Regression, SVM, Naive Bayes, LDA, Bagging, and GBDT.


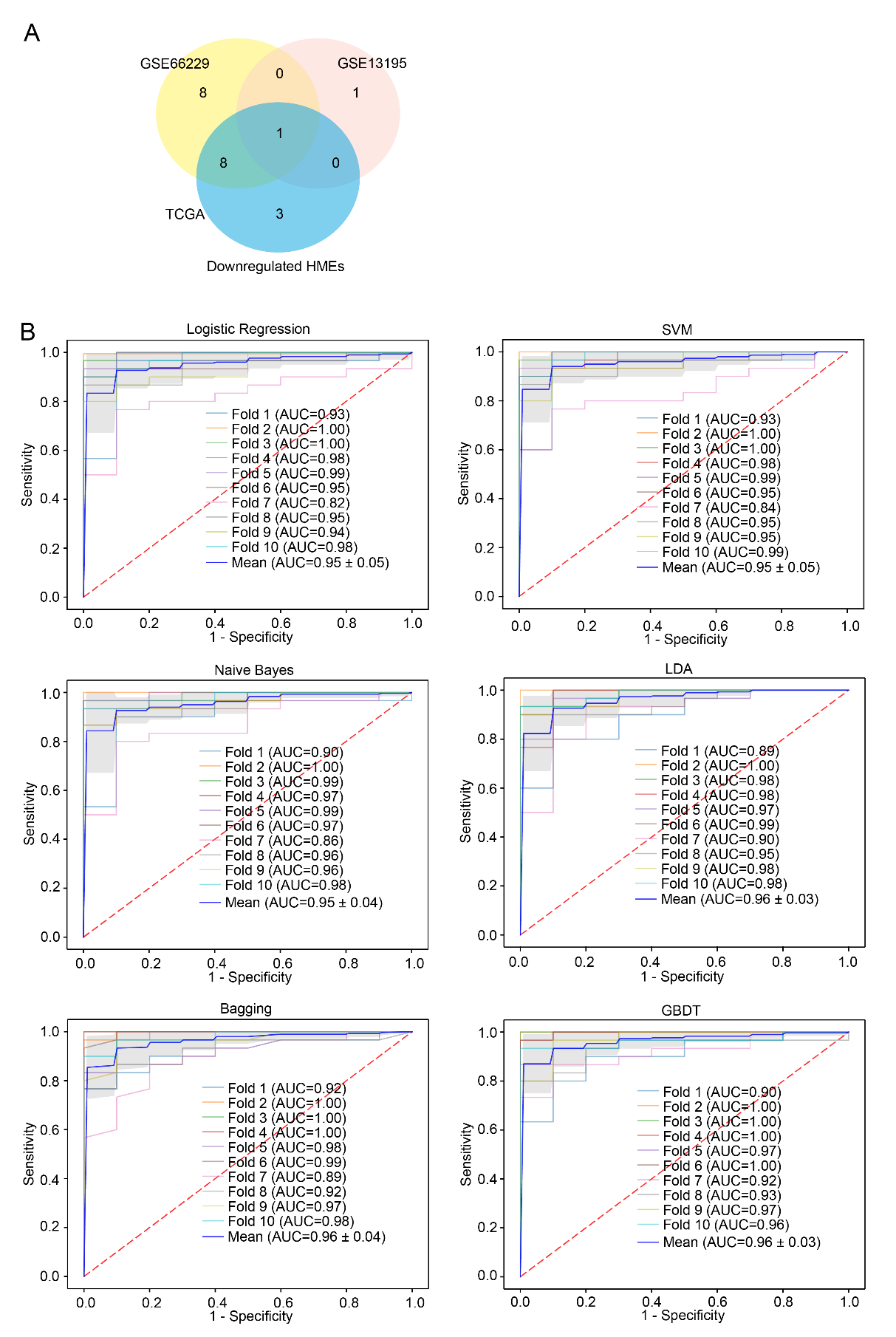


**Supplementary Figure 4.** **SMYD2 expression was associated with genomic alterations in cancers. (A)** Correlations between SMYD2 expression and tumor mutation burden (TMB) in multiple cancer types from TCGA dataset. **(B)** Co-occurrence and mutual exclusivity patterns of somatic mutations in high versus low SMYD2-expression group from TCGA dataset.


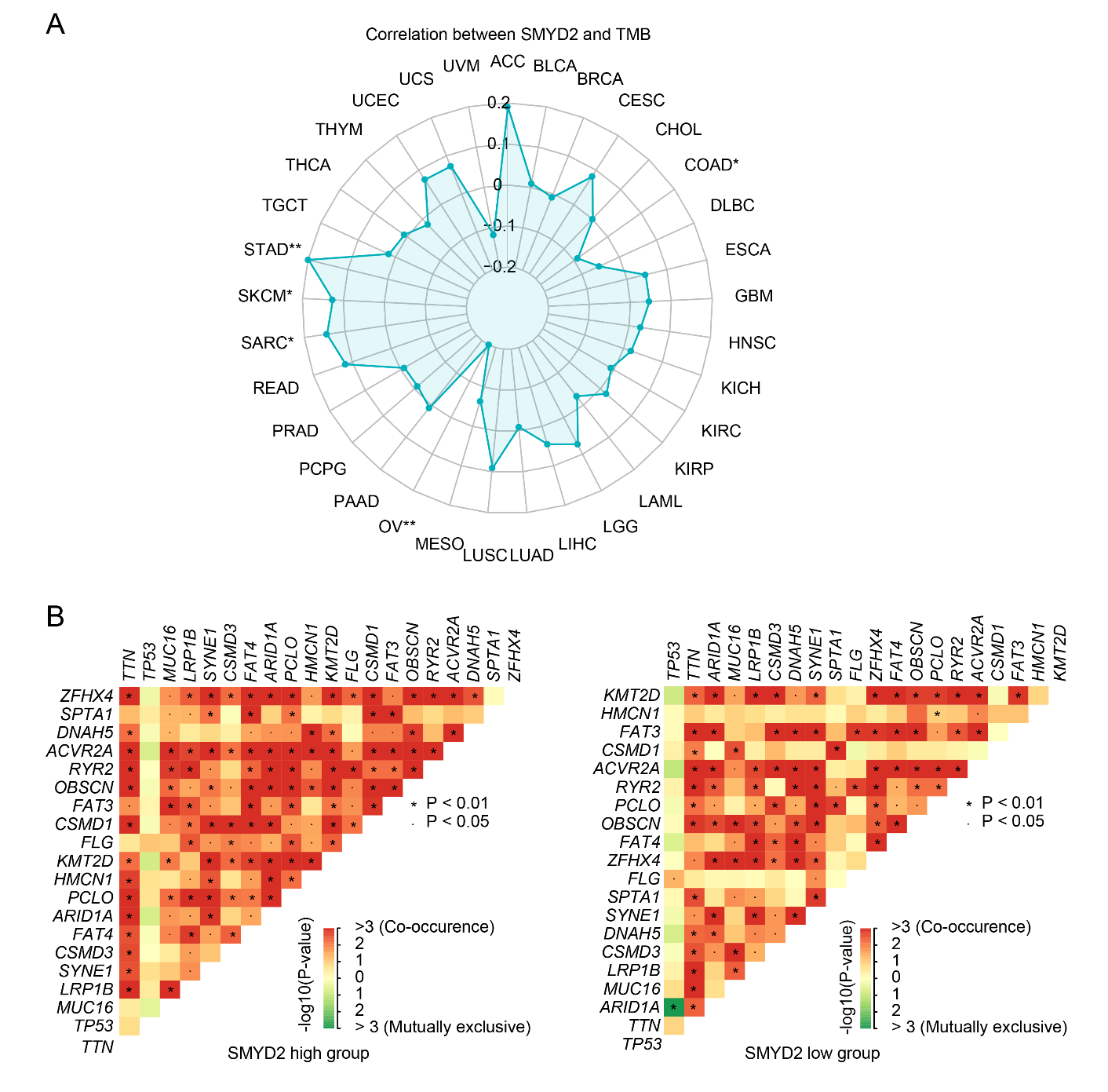


**Supplementary Figure 5.** **Potential pathways associated with SMYD2-downregulated genes. (A)** Venn diagram depicting common downregulated genes in the high SMYD2 group across the TCGA, GSE15459, GSE26253, GSE66229, and GSE84433. **(B and C)** Gene Ontology (B) and KEGG pathway (C) analyses of the 277 down-regulated genes identified in the high SMYD2 group.


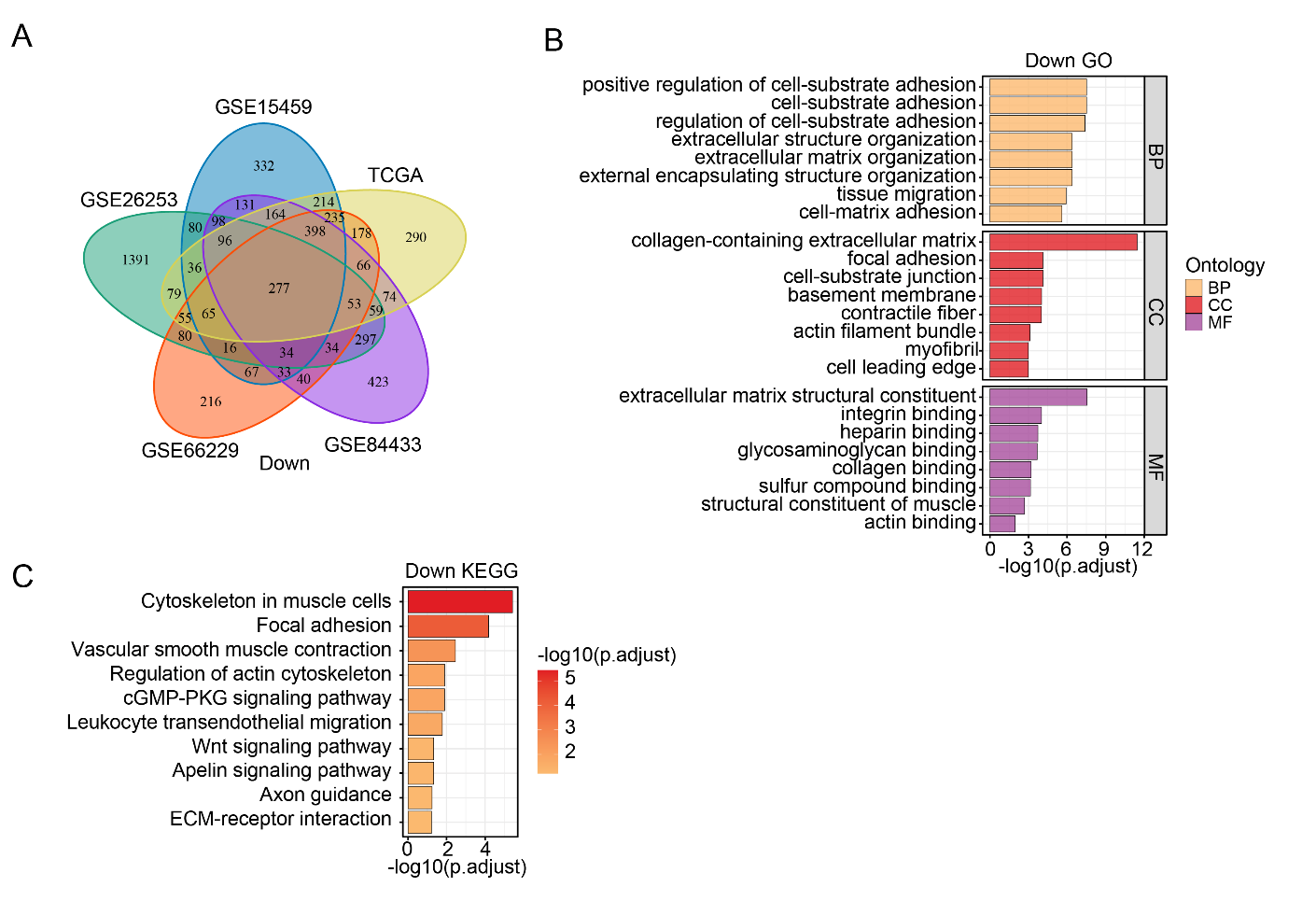


**Supplementary Figure 6.** ***H. pylori* infection promotes SMYD2 expression. (A)** The mRNA expression of SMYD2 in *H. pylori*-negative and *H. pylori*-positive human samples from GSE13195. ***P* < 0.01, Student’s *t*-test.


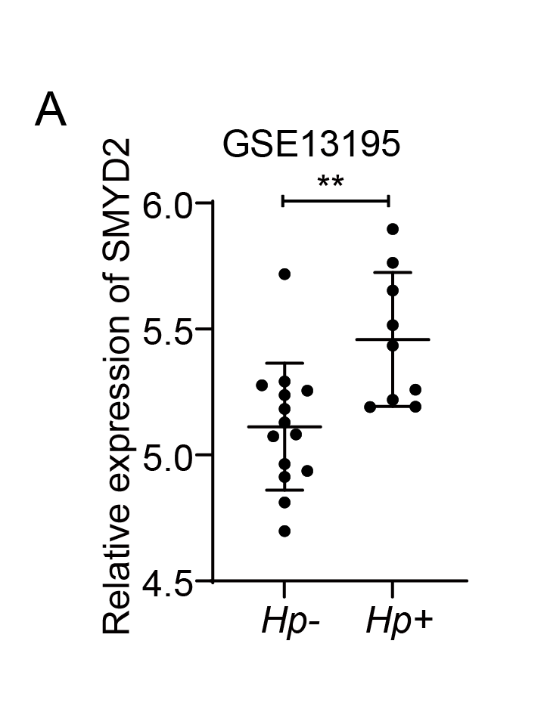


**Supplementary Table 1: 74 histone methyltransferases and histone demethylases.**

| **Number** | **Gene Name** | **Number** | **Gene Name** | **Number** | **Gene Name** | **Number** | **Gene Name** |
| --- | --- | --- | --- | --- | --- | --- | --- |
| 1 | KMT1A | 21 | KMT5A | 41 | PRMT8 | 61 | FBXL10 |
| 2 | KMT1B | 22 | KMT5B | 42 | PRMT9 | 62 | KIAA1718 |
| 3 | KMT1C | 23 | KMT5C | 43 | LSD1 | 63 | PHF2 |
| 4 | KMT1D | 24 | KMT6 | 44 | LSD2 | 64 | PHF8 |
| 5 | SETDB1 | 25 | KMT7 | 45 | JMJD1A | 65 | JARID1A |
| 6 | KMT1F | 26 | KMT8 | 46 | JMJD1B | 66 | JARID1B |
| 7 | KMT2A | 27 | SETMAR | 47 | JMJD1C | 67 | JARID1C |
| 8 | KMT2B | 28 | NSD2 | 48 | JMJD2A | 68 | JARID1D |
| 9 | KMT2C | 29 | NSD3 | 49 | JMJD2B | 69 | JARID2 |
| 10 | KMT2D | 30 | EZH1 | 50 | JMJD2C | 70 | HIF1AN |
| 11 | KMT2E | 31 | PRDM16 | 51 | JMJD2D | 71 | HSPBAP1 |
| 12 | KMT2F | 32 | SETD3 | 52 | JMJD3 | 72 | HR |
| 13 | KMT2G | 33 | SMYD5 | 53 | UTX | 73 | NO66 |
| 14 | KMT2H | 34 | PRMT1 | 54 | UTY | 74 | MINA |
| 15 | KMT3A | 35 | PRMT2 | 55 | JMJD4 |  |  |
| 16 | KMT3B | 36 | PRMT3 | 56 | JMJD5 |  |  |
| 17 | SMYD2 | 37 | PRMT4 | 57 | JMJD6 |  |  |
| 18 | KMT3D | 38 | PRMT5 | 58 | JMJD7 |  |  |
| 19 | KMT3E | 39 | PRMT6 | 59 | JMJD8 |  |  |
| 20 | KMT4 | 40 | PRMT7 | 60 | FBXL11 |  |  |

**Supplementary Table 2: Correlation between SMYD2 expression and clinicopathological characteristics in GC specimen.**

| **Clinicopathological Parameter** | **Number** | **SMYD2 expression** | | ***P*-value** |
| --- | --- | --- | --- | --- |
|  |  | Low (n=46) | High (n=48) |  |
| **Gender** |  |  |  | 0.957 |
| Male | 59 | 29 | 30 |  |
| Female | 35 | 17 | 18 |  |
| **Age (years)** |  |  |  | 0.22 |
| <65 | 35 | 20 | 15 |  |
| ≥65 | 59 | 26 | 33 |  |
| **Degree of differentiation** |  |  |  | 0.259 |
| Well | 21 | 8 | 13 |  |
| Moderate/poor | 73 | 38 | 35 |  |
| **Tumor diameter (cm)** |  |  |  | 0.774 |
| <5 | 32 | 15 | 17 |  |
| ≥5 | 62 | 31 | 31 |  |
| **Tumor site** |  |  |  | 0.812 |
| Gastric antrum | 34 | 18 | 16 |  |
| Gastric body | 44 | 21 | 23 |  |
| Gastric cardia | 16 | 7 | 9 |  |
| **Borrmann subtype** |  |  |  | 0.005 |
| Protrude type | 9 | 5 | 4 |  |
| Restricted ulcer type | 20 | 4 | 16 |  |
| Infiltrating ulcer type | 53 | 29 | 24 |  |
| Diffuse infiltrating type | 12 | 7 | 5 |  |
| **Pathological type** |  |  |  | 0.586 |
| Tubular adenocarcinoma | 78 | 38 | 40 |  |
| Mucinous adenocarcinoma | 6 | 4 | 2 |  |
| Signet-ring cell carcinoma | 10 | 4 | 6 |  |
| **TNM stage** |  |  |  | 0.414 |
| I+II | 35 | 19 | 16 |  |
| III+IV | 59 | 27 | 32 |  |
| **Depth of invasion** |  |  |  | 0.45 |
| T1+T2 | 15 | 6 | 9 |  |
| T3+T4 | 79 | 40 | 39 |  |
| **Lymph node metastasis** |  |  |  | 0.709 |
| Negative | 22 | 10 | 12 |  |
| Positive | 72 | 36 | 36 |  |
| **HER2** |  |  |  | 0.957 |
| Negative | 88 | 43 | 45 |  |
| Positive | 6 | 3 | 3 |  |
